# Design and immunogenic evaluation of multi-epitope vaccines for colorectal cancer: insights from molecular dynamics and *In-Vitro* studies

**DOI:** 10.3389/fonc.2025.1592072

**Published:** 2025-06-17

**Authors:** Peiwei Sun, Luolin Wang, Zhong Liu, Zhenglei Xu

**Affiliations:** ^1^ Department of General Surgery, Shenzhen University General Hospital/Shenzhen University Clinical Medical Academy, Shenzhen, China; ^2^ Department of Gastroenterology, Shenzhen People's Hospital (The First Affiliated Hospital, Southern University of Science and Technology; The Second Clinical Medical College, Jinan University), Shenzhen, Guangdong, China

**Keywords:** colorectal cancer, cytotoxic T lymphocyte, Dickkopf-like 1 protein, F-box protein 39, Opa-interacting protein 5, Toll-Like Receptor 4, *In-vitro* validation, molecular dynamics simulations

## Abstract

**Background:**

This study aimed to identify cytotoxic T lymphocyte (CTL)-specific epitopes from three tumor-associated antigens (TAAs)—Dickkopf-like 1 (DKKL1), F-box protein 39 (FBXO39), and Opa-interacting protein 5 (OIP5)—which are overexpressed in colorectal cancer (CRC), as potential candidates for CTL-mediated immunotherapy.

**Methods:**

The amino acid sequences of DKKL1, FBXO39, and OIP5 were analyzed to predict high-affinity CTL epitopes using the NetCTL server. Their antigenicity, allergenicity, conservation, and glycosylation potential were assessed for safety and effectiveness. Cross-reactivity and binding affinities were evaluated through molecular docking. Two multi-epitope vaccine constructs were designed incorporating the CTL epitopes, GM-CSF and IL-2 adjuvants, and a PADRE sequence. Docking studies with Toll-like receptor 4 (TLR-4) were performed. *In-vitro* assays using human peripheral blood mononuclear cells (PBMCs) were conducted to evaluate the immunogenicity of the vaccine constructs, focusing on cytokine release and T-cell activation. Additionally, molecular dynamics simulations were performed to assess the stability of peptide-HLA interactions.

**Results:**

High-affinity CTL-specific epitopes were successfully identified from DKKL1, FBXO39, and OIP5, showing strong binding potential to HLA class I molecules. The selected epitopes were predicted to be non-allergenic, non-glycosylated, and conserved across species. Molecular docking confirmed stable binding interactions between the epitopes and HLA alleles. *In-vitro* validation demonstrated that PBMCs stimulated with the multi-epitope vaccine constructs produced significant increase in cytokine levels, including IFN-γ and IL-2, indicative of robust CTL activation. Moreover, molecular dynamics simulations showed strong and stable binding affinities between the epitopes and HLA molecules, suggesting effective antigen presentation. Additionally, docking studies revealed strong binding affinities between the vaccine constructs and TLR-4, suggesting their potential to trigger a strong immune response.

**Conclusion:**

This study identified CTL-specific epitopes from DKKL1, FBXO39, and OIP5 as potential targets for colorectal cancer immunotherapy. The multi-epitope vaccine constructs exhibited significant immunogenic potential, providing a foundation for future clinical validation. These findings underscore the promise of these TAAs as key targets for CTL-based vaccine development in colorectal cancer.

## Introduction

Colorectal cancer (CRC) is among the most prevalent and fatal cancers globally, significantly contributing to cancer-related morbidity and mortality (1). This is the second leading cause of cancer-related deaths globally, with an estimated 881,000 deaths in 2018. Among CRC subtypes, colon cancer ranks fifth in mortality, responsible for 551,000 deaths (5.8% of all cancer deaths), while rectal cancer is tenth, causing 310,000 deaths (3.2% of total cancer deaths). The lifetime risk of dying from colon cancer is 0.66% for men and 0.44% for women, while for rectal cancer, the risk is 0.46% for men and 0.26% for women. The global age-standardized mortality rate for CRC is 8.9 per 100,000 people (2). Despite advancements in screening, diagnostics, and therapeutic options, the prognosis for advanced-stage CRC remains grim, with metastasis being a major cause of mortality (3). This underscores the urgent need for novel and more effective therapeutic strategies. Immunotherapy has emerged as a promising avenue, with peptide-based vaccines offering distinct advantages over other approaches. These vaccines are designed to elicit a robust CTL response, specifically targeting and eliminating cancer cells while minimizing off-target effects. Unlike monoclonal antibodies or checkpoint inhibitors, peptide-based vaccines can be tailored to induce long-lasting immune memory with fewer systemic side effects. Among the most promising tumor antigens for such vaccines are cancer-testis antigens (CTAs)—a unique class of tumor-associated antigens primarily expressed in male germ cells, particularly in the testis, while being absent in most normal tissues (4). However, their aberrant overexpression in various malignancies, including colorectal cancer, makes them attractive candidates for targeted immunotherapy. Notably, CTAs have been shown to play significant roles in tumor progression, invasion, and metastasis. Moreover, they are often associated with poor prognosis in cancer patients (5). More importantly, CTAs possess immunogenic properties, meaning they can trigger strong cell-mediated immune responses, particularly by activating CTLs. Some CTAs have also been shown to elicit humoral immunity, making them even more relevant for vaccine development (6, 7). Growing research has consistently shown the significant anti-tumour potential of vaccines that target cancer-testis antigens (CTAs) (8). Among the CTAs identified in colorectal cancer, NY-ESO-1, MAGE-A, LAGE-1, OIP5, TTK, PLU1, DKKL1, and FBXO39 are highly expressed, making them promising targets for immunotherapy (9). Among these CTAs: DKKL1, FBXO39, and OIP5 have emerged as particularly attractive targets for immunotherapy due to their specific overexpression in tumor tissues and roles in promoting oncogenesis. DKKL1 (Dickkopf-like 1) is known to modulate the Wnt/β-catenin signaling pathway, a critical regulator of cellular proliferation and differentiation in colorectal cancer, and its expression has been linked to enhanced tumor invasiveness and metastasis (10). FBXO39, an F-box protein family member, contributes to tumor cell survival by regulating proteasomal degradation pathways and has been implicated in promoting colorectal tumor progression and drug resistance (11). OIP5 (Opa-interacting protein 5), involved in centromere function and chromosomal stability, is highly expressed in several cancers including CRC and is associated with cell cycle progression, poor prognosis, and immune evasion (12). Importantly, these antigens have demonstrated the capacity to induce CTL responses, highlighting their potential for inclusion in cancer vaccine formulations. These antigens are not only selectively overexpressed in CRC cells but also capable of inducing immune responses, which makes them particularly valuable for epitope-based vaccine strategies. CTLs recognize and eliminate tumor cells by interacting with HLA class I molecules that present specific epitopes derived from CTAs. Identifying and designing these epitopes is crucial for developing effective peptide vaccines that can target colorectal cancer cells with precision. The advent of immuno-informatics tools has enabled the rapid and cost-effective prediction of HLA-restricted epitopes from tumor antigens, providing a streamlined pathway for epitope-based vaccine development. Unlike traditional experimental screening, which is time-consuming and resource-intensive, *in-silico* approaches allow for high-throughput analysis, prioritizing the most immunogenic candidates with greater precision (13). Epitope-based vaccines offer a significant advantage in that they are highly specific to tumor cells, thereby minimizing the risk of off-target effects and damage to healthy tissues (14). By selecting epitopes with a high binding affinity for HLA class I molecules, these vaccines can induce strong CD8+ T cell responses, which play a crucial role in the destruction of tumor cells. Computational tools such as NetCTL and IEDB are widely used to predict these epitopes, allowing researchers to focus on peptides that have the greatest potential to bind HLA molecules and stimulate an immune response (15). These tools offer high sensitivity and specificity in predicting HLA-restricted epitopes, significantly streamlining vaccine design. However, their accuracy can vary depending on the allele coverage and the quality of training data, necessitating experimental validation to confirm the immunogenic potential of predicted epitopes. In the present vaccine constructs, we incorporated two well-known adjuvants—GM-CSF and IL-2—due to their distinct immunostimulatory mechanisms. GM-CSF promotes dendritic cell maturation and antigen presentation, which enhances CD8+ T cell priming, while IL-2 supports T-cell proliferation and survival. Their inclusion was based on evidence from previous cancer vaccine studies demonstrating their capacity to amplify CTL responses and improve memory T-cell persistence. These properties make them suitable choices for enhancing vaccine immunogenicity in a tumor-specific context. This *in-silico* approach enables the rapid and cost-effective identification of potential vaccine candidates, which can later be validated experimentally. In colorectal cancer, CTAs such as DKKL1, FBXO39, and OIP5 hold great promise as therapeutic targets due to their overexpression and immunogenic properties. By incorporating adjuvants such as GM-CSF and IL-2, which are known to enhance immune responses, peptide vaccines targeting these CTAs can be optimized for greater efficacy. In view of this, the present study aims to identify lead CTL epitopes targeting DKKL1, FBXO39, and OIP5 CTAs for colorectal cancer.

## Materials and methods

### Amino acid sequences of DKKL1, FBXO39, and OIP5

The sequences for DKKL1, FBXO39, and OIP5 proteins were sourced from the NCBI protein database. A Protein BLAST search was conducted against the NCBI non-redundant protein database to identify homologous sequences. The sequences retrieved from this analysis were subjected to multiple sequence alignments using the COBALT to identify conserved regions and potential mutations. This step was crucial to understanding the sequence variability and conservation across different isoforms or species. The physicochemical properties of DKKL1, FBXO39, and OIP5 proteins were determined using the ProtParam tool https://web.expasy.org/protparam/ (16). Additionally, potential N-glycosylation sites in the proteins were identified using the NetNGlyc 1.0 server to ensure that selected epitopes were not located within glycosylated regions, which could potentially affect epitope processing and presentation (17).

### Epitope screening

The identification of CTL epitopes from DKKL1, FBXO39, and OIP5 was conducted using the NetCTL server-1.2 (18). This tool predicts CTL epitopes by integrating predictions of peptide binding to MHC class I molecules, proteasomal C-terminal cleavage, and TAP transport efficiency. The target HLA class I alleles were HLA-A02:01, HLA-A03:01, and HLA-B*07:02, which are commonly found in diverse human populations. Given the genetic heterogeneity of CRC across different ethnic groups, these alleles were selected to maximize epitope coverage across populations. However, further validation in ethnically diverse cohorts is necessary to confirm the broad applicability of the predicted epitopes.

### Filtering lead epitopes

A multi-step filtering process was employed to identify the most promising lead epitopes: Conservancy Analysis: The identified epitopes were assessed for their sequence conservation across different strains or isoforms of DKKL1, FBXO39, and OIP5. Only epitopes with 100% conservation were selected to ensure broad applicability and minimize the risk of immune escape due to genetic variation in the target proteins.

Antigenicity Assessment: The likelihood of the epitopes being recognized as antigens by the immune system was determined using VaxiJen 2. o server (19), an alignment-independent tool that predicts the antigenicity of peptides based on their physicochemical properties. The threshold for determining the antigenicity score was kept at default i.e. 0.4.

Allergenicity Prediction: To avoid adverse allergic reactions, the shortlisted epitopes were screened for allergenicity using the AllerTOP v2.0 server (20). Non-allergenic epitopes were selected for further analysis.

Glycosylation Site Analysis: The selected epitopes were analyzed for their location concerning glycosylation sites using the NetNGlyc 1.0 server (21). Only epitopes that were not located within predicted glycosylation sites were chosen, as glycosylation can interfere with epitope processing and presentation to T cells.

### Molecular docking with HLA alleles

The final set of filtered epitopes was subjected to molecular docking with the target HLA class I alleles (HLA-A02:01, HLA-A03:01, and HLA-B*07:02) to analyze their binding affinity and patterns. The structural data for the HLA alleles were sourced from the Protein Data Bank. The ClusPro server was sourced to undertake molecular docking analysis (22), which allows for the evaluation of peptide-HLA interactions by providing docking scores and visualizing the binding patterns. The quality of the binding interactions was assessed based on parameters such as binding energy and the number of hydrogen bonds.

### Multi-CTL epitope chain construction

Two multi-epitope vaccine constructs were developed by connecting the selected CTL epitopes with AAY linkers. This linker sequence was chosen based on previous studies that demonstrated its effectiveness in maintaining epitope stability and facilitating proper processing and presentation within the vaccine construct. AAY linkers are known to enhance proteasomal cleavage and support efficient antigen presentation, making them a preferred choice in multi-epitope vaccine design. While alternative linkers exist, our selection was informed by established literature on their advantages. To further improve immunogenicity, a PADRE sequence was added to the vaccine constructs to stimulate robust activation of T-helper cells, which plays a crucial role in enhancing the overall immune response. The PADRE sequence was linked to the chain using an EAAAK linker to maintain spatial separation and reduce steric hindrance. Additionally, two adjuvants, Granulocyte-Macrophage Colony-Stimulating Factor (GM-CSF) and Interleukin-2 (IL-2), were fused to the N-terminal of the epitope chain via an EAAAK linker. This strategy aims to amplify the immune response by leveraging the immune-boosting properties of these adjuvants. The constructs were modeled using the Robetta server, followed by refinement using the GalaxyRefine server (23) to enhance overall structural quality. The refined models were validated through Ramachandran plot analysis to confirm their structural integrity, ensuring proper folding and stability.

### Immune simulation

The vaccine constructs were subjected to a C-ImmSim server for *in-silico* immune simulation (24) to predict the immune response they might elicit. The protocol followed for immune simulation was based on the methodology described by Chauhan et al. (25). The simulation aimed to analyze the CTL, helper T-cell, and memory cell responses generated by the vaccine constructs over time, providing insights into their potential efficacy.

### Molecular docking with TLR-4

To assess the interaction between the vaccine constructs and the immune system, both constructs were docked with Toll-like Receptor 4 (TLR-4), a key receptor involved in the innate immune response. The docking was conducted using the ClusPro server, allowing the evaluation of binding affinities and patterns. High binding affinity to TLR-4 would suggest that the vaccine constructs could effectively activate the immune response, enhancing the immunogenicity and effectiveness of the designed vaccine. Docking parameters included: rigid-body docking mode, 1000 initial models, and clustering with a 9 Å Cα RMSD threshold. The evaluation criteria for docking included balanced energy scores, electrostatic and hydrophobic contributions, number of hydrogen bonds, and Van der Waals + electrostatic interaction energies. ClusPro’s top-ranked cluster models (based on population size and lowest energy scores) were selected for further analysis. Molecular visualization was performed using PyMOL.

### *In-Vitro* validation of epitope immunogenicity

To evaluate the immunogenic potential of the selected epitopes, an *in vitro* assay was conducted using human peripheral blood mononuclear cells (PBMCs). PBMCs were isolated from healthy donors through density gradient centrifugation (e.g., Ficoll-Paque) and cultured in RPMI-1640 medium supplemented with 10% fetal bovine serum (FBS), 2 mM L-glutamine, 1% penicillin-streptomycin, and 1% non-essential amino acids. Cells were incubated at 37°C in a humidified atmosphere containing 5% CO_2_. The PBMCs were stimulated with synthetic peptides corresponding to the selected epitopes at concentrations ranging from 5 to 20 µg/mL. After 48–72 hours of incubation. Supernatants were collected to assess cytokine secretion profiles. To ensure the accuracy of cytokine quantification, both positive and negative controls were included. The positive control consisted of PBMCs stimulated with a known immunogenic peptide, while the negative control involved unstimulated PBMCs. Phytohemagglutinin (PHA; Sigma-Aldrich, Cat. No. L8754) at a concentration of 10 µg/mL was used as a positive control to validate the responsiveness of PBMCs to stimulation. The levels of IFN-γ (ELISA MAX™ Deluxe Set, Cat. No. 430104), IL-2 (ELISA MAX™ Deluxe Set, Cat. No. 431807), and TNF-α (ELISA MAX™ Deluxe Set, Cat. No. 430204) were quantified using enzyme-linked immunosorbent assay (ELISA) kits according to the manufacturer’s instructions. The results were analyzed to determine the ability of the epitopes to induce T-cell activation and cytokine production. All *in-vitro* experiments were performed in triplicate (n = 3 independent donors), and each measurement was repeated at least three times to ensure reproducibility. Cytokine levels were reported as mean ± standard deviation (SD). Statistical analysis was conducted using GraphPad Prism v9.0 software. A p-value < 0.05 was considered statistically significant.

### Molecular dynamics simulations

To further assess the stability of peptide-HLA complexes, molecular dynamics (MD) simulations were performed using GROMACS 2022. The docked complexes were solvated in a TIP3P water box, neutralized with counterions, and subjected to energy minimization using the steepest descent algorithm. The system was equilibrated under NVT and NPT conditions for 100 ps each, followed by a 50 ns production run at 310 K using a Berendsen thermostat. Root-mean-square deviation (RMSD), root-mean-square fluctuation (RMSF), and hydrogen bonding analyses were conducted to evaluate the structural stability and binding dynamics of the epitope-HLA interactions.

## Results

### Amino acid sequences and conservation analysis

The protein sequences of DKKL1, FBXO39, and OIP5 were successfully obtained from the NCBI protein database. Following this, an extensive protein BLAST search was performed, yielding multiple homologous sequences. These sequences were subjected to multiple sequence alignment (MSA) using the COBALT tool, revealing conserved regions (**Supplementary Figure S1**). Several regions within each protein showed high conservation, indicating their potential as stable epitope targets. The physicochemical characteristics of these proteins were analyzed using ProtParam, revealing key characteristics such as molecular weight, isoelectric points, and stability indices (**Supplementary Table S1**). The NetNGlyc 1.0 server identified several N-glycosylation sites in each protein sequence (**Supplementary Figure S2**). By identifying these sites, we ensured that our selected epitopes were not located within or near glycosylation regions, thus avoiding potential interference with epitope processing and presentation.

### Filtering lead epitopes

**Figure 1:**
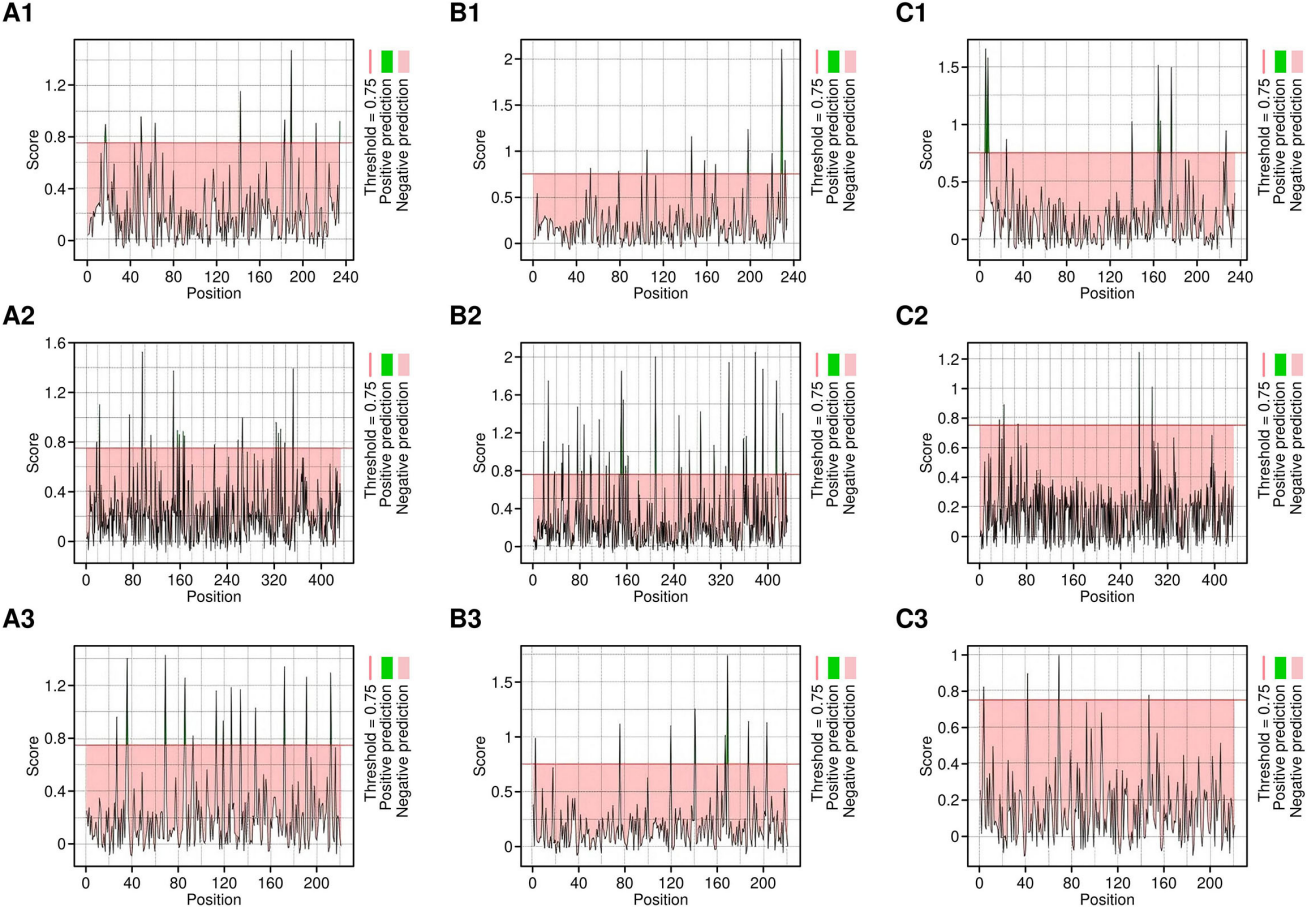
CTL epitopes predicted by NetCTL server. A1-C1 are the epitopes predicted targeting HLA-A*02:01 allele, A2-C2 are the epitopes predicted targeting the HLA-A*03:01 allele and, A3-C3 are the epitopes predicted targeting the HLA-B*07:02 allele, respectively.

CTL epitopes were predicted for each of the target proteins using the NetCTL 1.2 server, with particular attention to their ability to interact with HLA class I alleles (HLA-A02:01, HLA-A03:01, and HLA-B*07:02) (**Figure 1**). Only the epitopes with a threshold of 0.75 were included for further screening.

The filtering process was comprehensive and meticulous as follows:

Conservancy Analysis: The selected epitopes were evaluated for conservation across the aligned sequences. Epitopes with 100% conservation were prioritized, resulting in epitopes each for DKKL1, FBXO39, and OIP5 that were completely conserved. This high level of conservancy suggests that these epitopes are less likely to undergo mutations that could lead to immune escape.

Antigenicity Assessment: The conserved epitopes were assessed for their antigenicity using the VaxiJen v2.0 server. Epitopes with scores exceeding the threshold of 0.4 were chosen, suggesting a high likelihood of being recognized by the immune system as potential antigens.

Allergenicity Prediction: These antigenic epitopes were further screened for allergenicity using AllerTOP v2.0. The analysis revealed that the epitopes were non-allergenic, which significantly reduced the likelihood of adverse immune reactions. Importantly, the selection of non-allergenic epitopes does not compromise the vaccine’s ability to generate a robust immune response. On the contrary, the absence of allergenic properties ensures that the immune system’s focus remains on the targeted tumor antigens, enhancing the specificity and effectiveness of the immune activation.

Glycosylation Site Analysis: Finally, the selected epitopes were cross-referenced with glycosylation sites identified earlier. The epitopes which were found to be located outside of glycosylation sites were only selected, indicating they would be effectively processed and presented by the immune system.

The finalized epitopes were high binders to HLA molecules as revealed by the NetCTL 1.2 server as we selected only those epitopes which were having high binding scores. In addition, they were conserved, immunogenic, non-allergenic and located in non-glycosylation sites. Based on these filtering criteria, 3 epitopes for DKKL1, 10 for FBXO39, and 2 for OIP5 were shortlisted (**Table 1**).

**Table 1:** Finally screened out lead CTL epitopes.

Protein	Epitope position (Code)	Epitope sequence	Prediction Score	Vaxijen Score
DKK1	189 (E8)	WLSEKRHRL	1.4744	0.61
	50 (E2)	SLLQGFSRL	0.75	0.74
	232 (E12)	LLYILRPSR	0.8971	0.97
FBXO39	96 (E4)	FMNPYNAVL	1.5244	1.4
	23 (E1)	CLCRVFWWL	1.0992	1.65
	74 (E3)	EVESAVWYV	1.0189	0.58
	332 (E13)	LLPTFRHTL	0.9024	0.61
	219 (E11)	TMSTFHNLV	0.7752	1.41
	334 (E14)	PTFRHTLQK	1.9403	0.74
	392 (E15)	RQCALRVFK	1.8679	1.43
	98 (E5)	NPYNAVLTK	0.9561	1.21
	99 (E6)	PYNAVLTKK	0.8836	0.81
	125 (E7)	RLKSLSIQY	0.8425	1.21
OIP5	191 (E9)	PLSEKIAEL	1.2603	0.72
	203 (E10)	IVLTHNRLK	1.1279	0.94

The epitopes amino acid sequence, position, NetCTL score and Vaxijen score is represented.

### Molecular docking with HLA alleles

**Figure 2:**
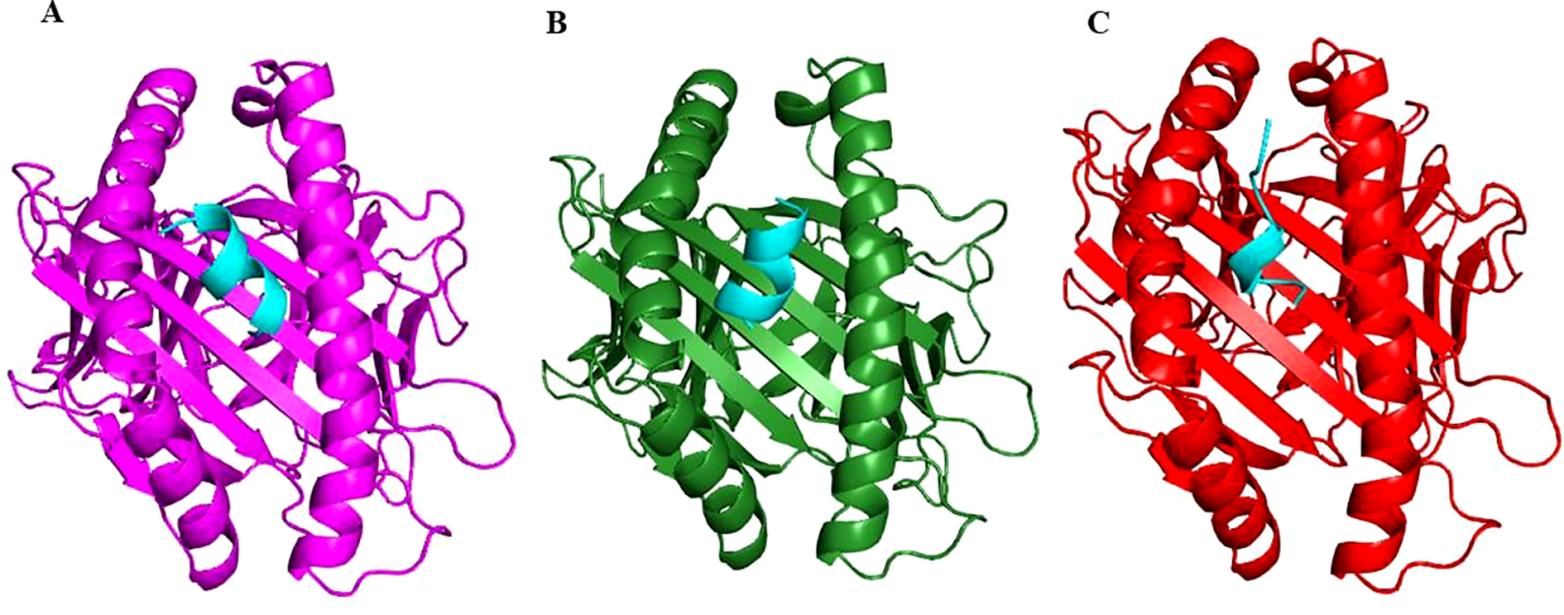
Cartoon representation showing binding of Epitope E1 (cyan) in the active site of HLA Class I alleles: HLA-A*02:01 (magenta) **(A)**, HLA-A*03:01 (dark green) **(B)** and HLA-B*07:02 (red) **(C)**.

The finalized epitopes were docked with the target HLA class I alleles using the ClusPro server. Docking analysis showed that all selected epitopes exhibited strong binding affinities, with negative binding energies indicative of stable complexes (**Table 2**). Visual analysis of the docking interactions revealed multiple hydrogen bonds and hydrophobic contacts between the epitopes and the binding grooves of the HLA molecules (**Figure 2**). The overall docking results suggested their high potential to be effectively presented to cytotoxic T cells.

**Table 2:** Docking scores of the lead epitopes with HLA Class I alleles.

Amino acid	Balanced	Electrostatic favoured	Hydrophobic-favoured	VdW+Elec
HLA-A*:0201				
E1	-1043.3	-1027.4	-1764.9	-128.7
E2	-651.1	-743.3	-1031.6	-128.5
E3	-819.1	-861.2	-1217.9	-122.4
E4	-684.2	-682.8	-1054.8	-103.9
E5	-628.2	-630.0	-892.8	-154.0
E6	-547.0	-579.6	-727.1	-124.0
E7	-633.0	-638.2	-901.1	-143.9
E8	-651.7	-711.7	-1020.6	-144.5
E9	-583.9	-628.1	-801.1	-132.0
E10	-682.1	-726.5	-1020.3	-134.9
E11	-731.0	-733.3	-1172.5	-92.4
E12	-762.7	-748.3	-1085.8	-164.1
E13	-754.2	-754.3	-1201.9	-114.9
E14	-626.6	-626.2	-967.4	-135.0
E15	-746.2	-744.8	-1234.1	-154.4
HLA-A*:0301				
E1	-942	-981.7	-1607.2	-143.2
E2	-641.4	-675.2	-993.5	-126.2
E3	-718.0	-695.0	-1015.3	-109.4
E4	-643.1	-652.8	-1060.0	-87.8
E5	-594.8	-613.7	-778.7	-141.5
E6	-474.5	-517.9	-613.9	-146.6
E7	-542.7	-597.5	-752.5	-160.5
E8	-650.0	-709.3	-891.1	-161.3
E9	-469.6	-471.6	--609.4	-114.1
E10	-724.4	-749.8	-1014.8	-151.9
E11	-655.0	-660.5	-1110.0	-99.8
E12	-758.1	-816.5	-1097.1	-193.2
E13	-628.6	-704.8	-1010.2	-144.4
E14	-605.7	-660.2	-828.1	-163.1
E15	-713.8	-769.2	-1088.4	-187.4
HLA-B*07:02				
E1	-1106.3	-1025.0	-1787.1	-148.4
E2	-718.5	-741.2	-1083.8	-146.3
E3	-761.9	-751.9	-1120.2	-106.5
E4	-746.5	-748.2	-1170.7	-92.7
E5	-650.1	-643.3	-884.8	-158.3
E6	-520.8	-562.8	-605.1	-168.6
E7	-652.8	-671.3	-890.7	-162.3
E8	-739.7	-775.0	-974.0	-161.1
E9	-567.5	-580.3	-748.6	-135.5
E10	-778.7	-813.2	-1134.9	-167.3
E11	-802.7	-807.1	-1217.3	-99.5
E12	-789.6	-836.3	-1231.6	-184.7
E13	-805.8	-841.4	-1201.3	-132.9
E14	-690.5	-737.2	-951.9	-161.6
E15	-725.1	-774.3	-1119.8	-192.2
VACCINE-1	-1346.5	-1472.6	-1596.2	-261.7
VACCINE-2	-1116.4	-1218.2	-1203.9	-259.8

### Multi-CTL epitope chain construction

**Figure 3:**
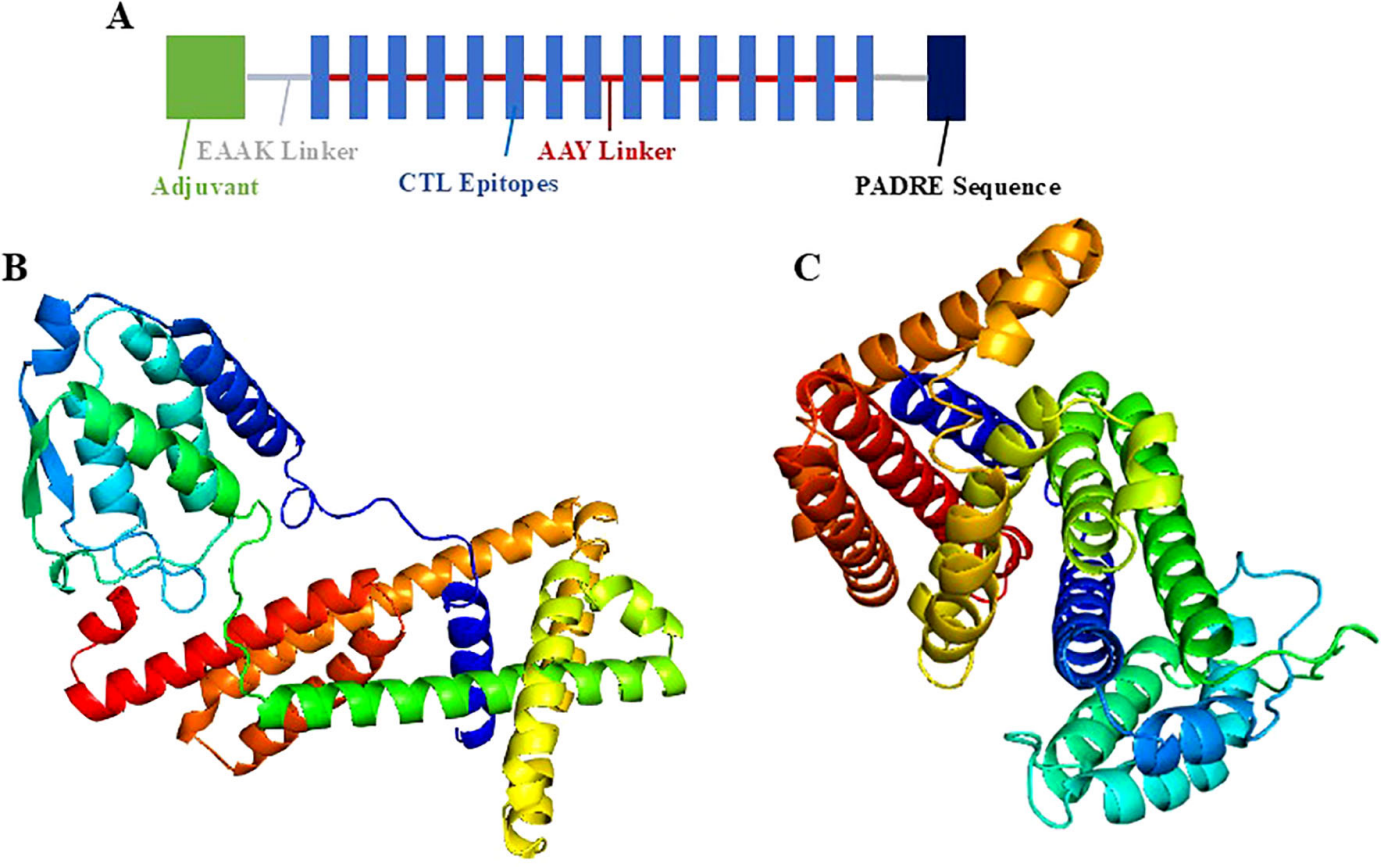
Modelled vaccine constructs. A- Overall layout of the vaccine construct. B&C- 3D models of Vaccine 1 containing GM-CSF as adjuvant and Vaccine 2 containing IL-2 as adjuvant.

**Figure 4:**
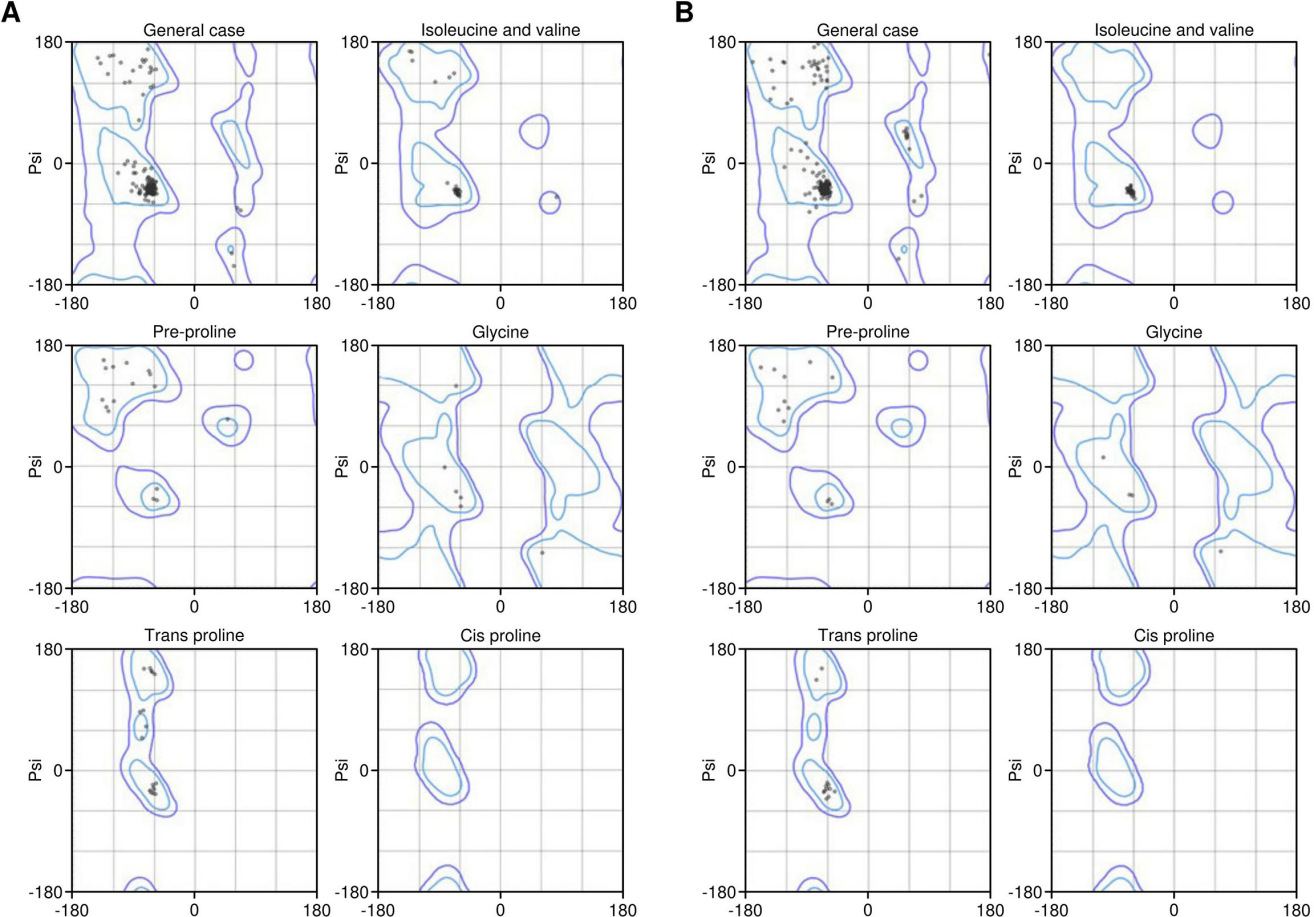
Ramachandran plot results of Vaccine 1 **(A)** and Vaccine 2 **(B)** constructs. The percentage of residues in the allowed region was 100% in both models.

The identified lead epitopes were assembled into two multi-epitope constructs. The first construct incorporated GM-CSF as an adjuvant, while the second used IL-2. Each construct included the identified CTL epitopes linked by AAY linkers and a PADRE sequence linked via an EAAAK linker (**Figure 3A**). The constructs were successfully modelled using the Robetta server, with structural refinement performed using the GalaxyRefine server (**Figure 3B, C**). The Ramachandran plot analysis revealed that 100% of residues were located in the allowed regions, indicating a high-quality model (**Figure 4**). This suggests that the constructed vaccines have a stable conformation.

### Immune simulation

**Figure 5:**
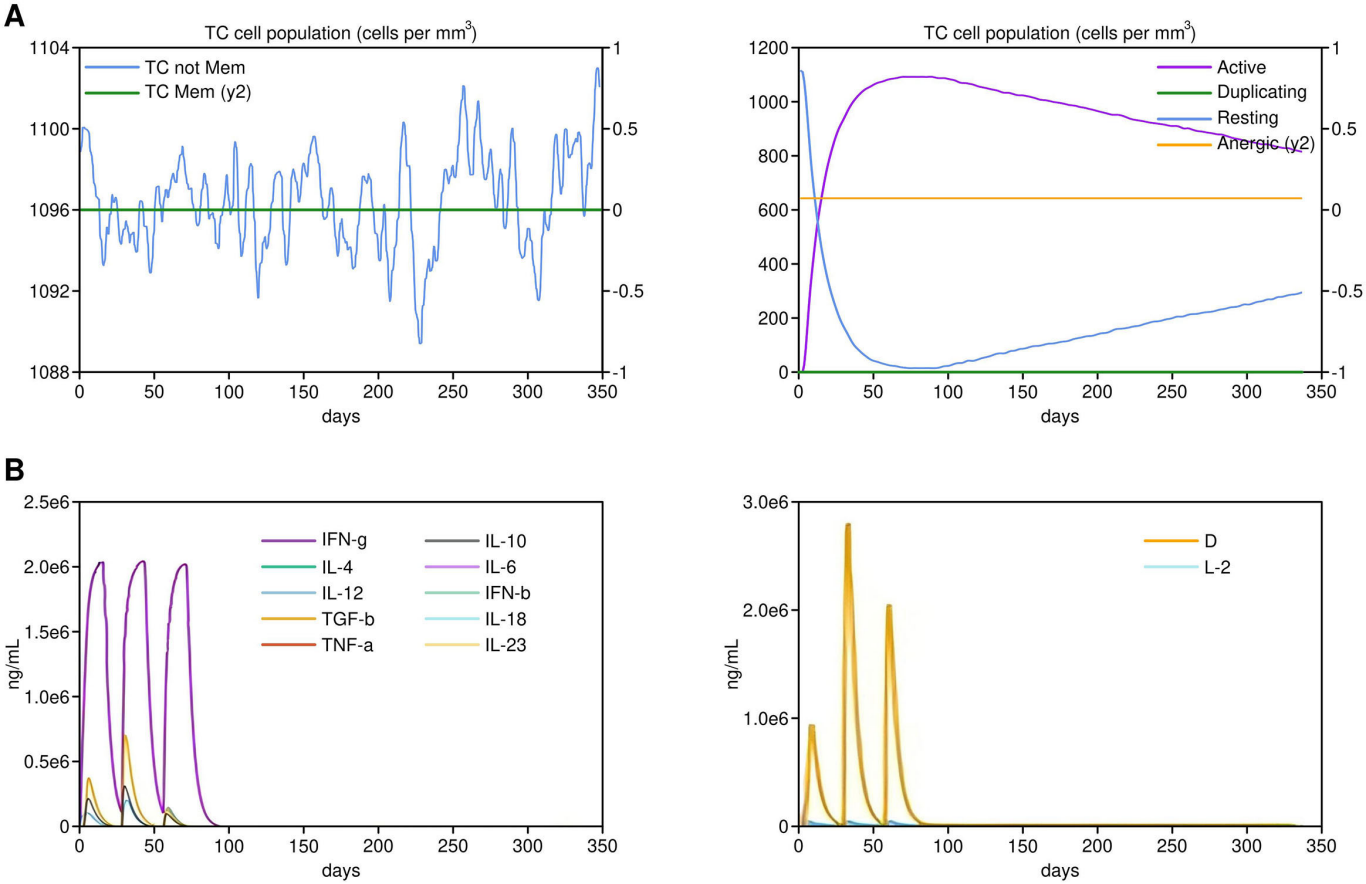
*In-silico* immune simulation results by the Vaccine 1 containing GM-CSF as an adjuvant.

*In-silico* immune simulations performed using the C-ImmSim server demonstrated a robust immune response. Both vaccine constructs generated a significant CTL response, with high levels of IFN-γ, IL-2, and memory T cells, indicating strong cellular immunity. Notably, the construct containing GM-CSF as an adjuvant elicited a higher frequency of CTLs and a more prolonged memory response compared to the construct with IL-2, highlighting its potential as an effective cancer vaccine (**Figure 5**). While cytokine secretion was assessed as a marker of immune activation, future experiments will include functional assays, such as cytotoxicity assays, to directly confirm the CTL-mediated tumor cell killing activity.

### Molecular docking with TLR-4

**Figure 6:**
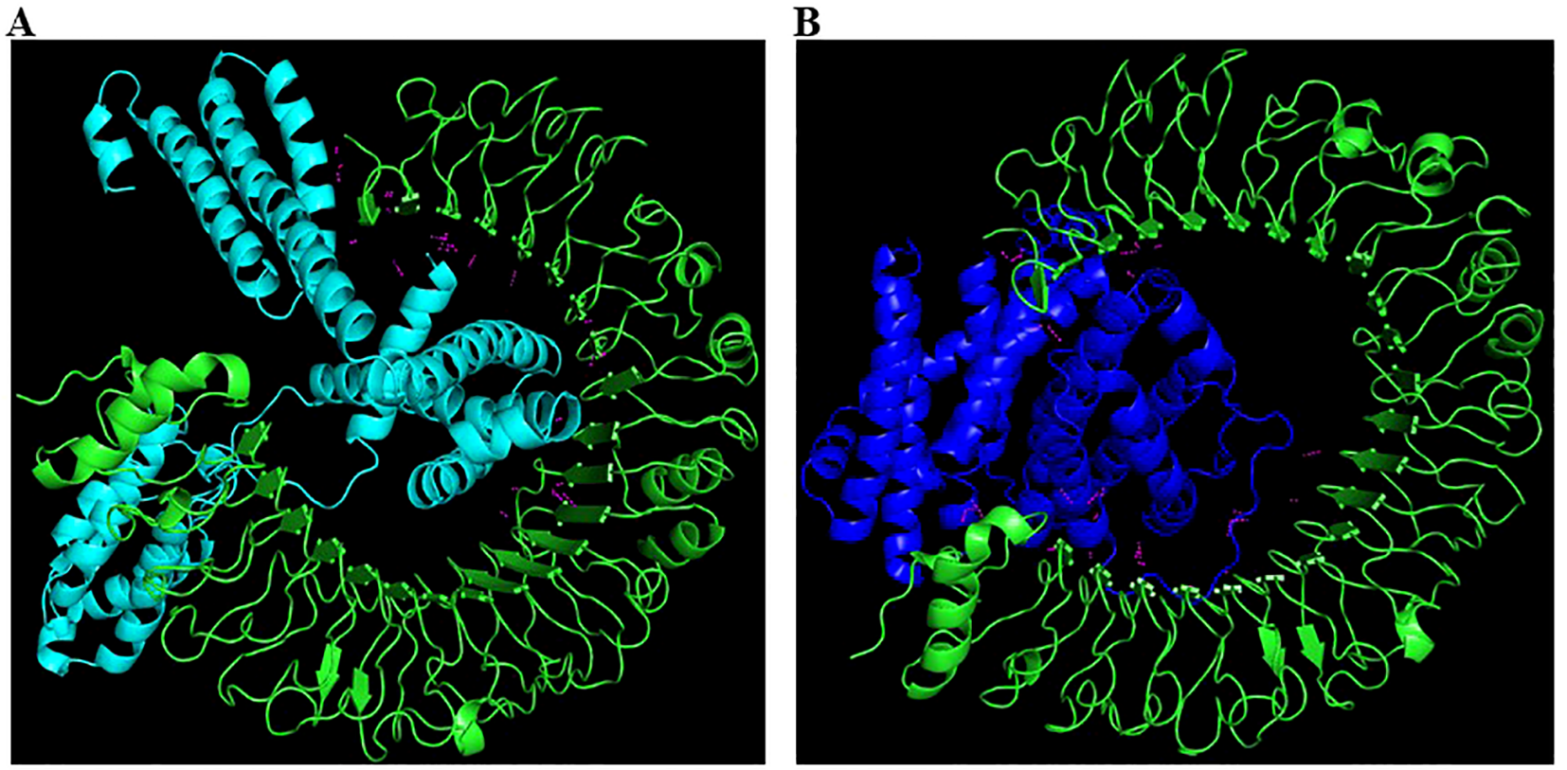
Interaction analysis of Vaccine 1 and Vaccine 2 constructs with TLR-4. A: Vaccine 1 (cyan colour) docked with TLR-4 (green colour). Note that the vaccine is inserted deep into TLR-4 forming numerous H-bonds (magenta). B: Vaccine 2 (dark blue colour) docked with TLR-4 (green colour). The H-bonds formed are represented in magenta colour.

The molecular docking analysis of both vaccine constructs with TLR-4 using the ClusPro server showed strong binding affinities. The GM-CSF-containing construct demonstrated a slightly higher binding affinity with TLR-4 i.e. -1346.5 kD than the IL-2-containing construct which showed -1116.4 kD balanced energy (**Table 1**). Detailed visualization of the docking complexes revealed multiple hydrogen bonds and hydrophobic interactions at the binding interface, indicating stable and effective binding to TLR-4 (**Figure 6**). This indicates that the vaccine constructs are likely to stimulate the innate immune response, thereby improving the overall immunogenicity of the vaccines.

### *In-Vitro* validation of epitope immunogenicity

PBMCs stimulated with the selected multi-epitope vaccine constructs exhibited a dose-dependent increase in cytokine secretion. IFN-γ levels were significantly elevated in cultures treated with 10 µg/mL (*p* < 0.01) and 20 µg/mL (*p* < 0.001) peptide concentrations, with peak secretion at 20 µg/mL. IL-2 secretion also showed a significant increase at 10 µg/mL (*p* < 0.01) and 20 µg/mL (*p* < 0.001). TNF-α levels demonstrated a moderate but statistically significant elevation at 20 µg/mL (*p* < 0.05), confirming robust CD8+ T cell activation (**Table 3**).

**Table 3:** Summary of cytokine secretion levels.

Peptide Concentration (µg/mL)	IFN-γ (pg/mL)	IL-2 (pg/mL)	TNF-α (pg/mL)
0 (Control)	12.3 ± 2.1	8.9 ± 1.8	5.6 ± 1.2
5	45.7 ± 5.3	23.5 ± 3.6	14.8 ± 2.7
10	89.2 ± 7.6^**^	47.8 ± 4.9^**^	29.3 ± 3.4
20	132.5 ± 9.1^***^	78.6 ± 6.8^***^	41.2 ± 4.1^*^

Data are expressed as mean ± standard deviation. Asterisks indicate statistically significant differences compared to the control group (0 µg/mL): *p* < 0.05 (*), *p* < 0.01 (**), *p* < 0.001 (***), determined by Student’s t-test.

### Molecular dynamics simulations

Molecular dynamics simulations revealed stable interactions between the selected epitopes and HLA class I alleles. RMSD analysis indicated minimal structural fluctuations, with values stabilizing within the 2.5–3.2 Å range, suggesting strong binding affinity. RMSF analysis further confirmed the stability of key binding residues, particularly in the peptide-binding groove. Hydrogen bonding analysis demonstrated persistent interactions between epitope residues and the HLA molecules, with an average of 4–6 hydrogen bonds maintained throughout the simulation. These results collectively suggest that the selected epitopes exhibit strong immunogenic properties and stable binding to HLA class I molecules, supporting their potential as viable vaccine candidates. Let me know if you need further refinements.

## Discussion

The development of a multi-epitope vaccine for CRC is a promising approach given the limitations of current treatment modalities, including surgery, chemotherapy, and immunotherapy (26). Multi-epitope vaccines have shown the potential to elicit robust immune responses by targeting multiple antigenic sites, thereby reducing the chances of immune evasion by tumor cells (27). In this study, we focused on three proteins: DKKL1, FBXO39, and OIP5, which are crucial for CRC pathogenesis and progression. DKKL1 is a key component of the Wnt/β-catenin signaling pathway, which is frequently dysregulated in CRC. Previous research has shown that DKKL1 contributes to tumor cell invasion and metastasis, highlighting its importance in cancer progression (28). Moreover, the downregulation of DKKL1 has been linked to the progression of colon cancer (29). By targeting DKKL1, our study aims to exploit the immunogenic potential of conserved epitopes within this protein, which could elicit an effective immunity against CRC cells expressing this antigen. FBXO39, part of the F-box protein family, is involved in tumorigenesis and has been associated with the development and progression of multiple cancers, including CRC (30). Recent studies have shown that FBXO39 can activate the Wnt/β-catenin pathway, thereby contributing to the proliferation and invasion of CRC cells (31). Our study identified multiple conserved epitopes within FBXO39, indicating that it is a viable target for CTL-based immunotherapy.

OIP5, known for its role in cell cycle regulation, is overexpressed in various cancers and has been associated with poor prognosis in CRC patients (32). OIP5 contributes to genomic instability and tumor growth, making it a potential target for vaccine development (33). By incorporating conserved epitopes from OIP5, we aim to elicit a specific cytotoxic T-cell response capable of recognizing and eliminating CRC cells. The amino acid sequence and conservation analysis conducted in our study revealed several highly conserved regions within DKKL1, FBXO39, and OIP5. Conservation of epitopes is a key feature in vaccine design as it increases the likelihood of broad population coverage and reduces the potential for immune escape. In contrast to neoantigen-based vaccines, which focus on targeting mutation-specific antigens unique to individual tumors, our multi-epitope vaccine targets conserved tumor-associated antigens (DKKL1, FBXO39, and OIP5) that are widely expressed across CRC patients. This broader applicability of conserved epitopes may allow for a more efficient and accessible vaccine strategy, potentially eliminating the need for personalized tumor profiling, a limitation of neoantigen-based approaches. Our study ensured that the selected epitopes were not located within glycosylation sites, which could interfere with proper antigen processing and presentation, thus optimizing their immunogenicity. The immunogenicity of the selected epitopes was confirmed through antigenicity and allergenicity assessments, ensuring that they were both immunostimulatory and safe. These epitopes were also shown to bind effectively to multiple HLA alleles (HLA-A02:01, HLA-A03:01, and HLA-B*07:02), indicating their potential to be presented to a broad population. Our results are consistent with other studies that have developed multi-epitope vaccines targeting cancer antigens such as CEA, MUC1, and EGFR in CRC (34). Molecular docking studies revealed strong binding affinities of the finalized epitopes to HLA class I alleles, highlighting their potential for effective presentation to cytotoxic T cells. The robust binding interactions observed in our docking analysis further validate the stability and efficacy of these epitopes in eliciting a T-cell response.

In designing the multi-epitope vaccine, we included immunoadjuvants GM-CSF and IL-2 to bolster the immune response. GM-CSF is commonly utilized as an adjuvant in cancer vaccines because of its capacity to stimulate dendritic cell maturation and enhance T-cell activation (35, 36). The inclusion of GM-CSF in our vaccine construct elicited a higher frequency of CTLs and a prolonged memory response, suggesting its superior efficacy in this context. Furthermore, the molecular docking of the multi-epitope vaccine constructs with TLR-4 demonstrated strong binding affinities, particularly with the GM-CSF-containing construct. TLR-4 engagement is crucial for activating innate immunity and enhancing the adaptive immune response, indicating that our vaccine constructs have the potential to trigger a comprehensive anti-tumor response (37). Our findings suggest that the multi-epitope vaccine constructs targeting DKKL1, FBXO39, and OIP5 have significant potential to induce a targeted immune response against CRC. While the combination of conserved, immunogenic, and non-allergenic epitopes with potent adjuvants, such as GM-CSF and IL-2, is expected to enhance the immune response, we recognize the potential for immunodominance, where certain epitopes may dominate the immune response. Future studies will explore optimizing the balance of epitope representation and adjuvant use to ensure a well-rounded immune response that targets all relevant epitopes.

Several previous studies have explored multi-epitope vaccines and CTL-targeted strategies in cancer and other malignancies. For instance, Freiberger et al. (2023) designed a multi-epitope peptide vaccine targeting MAGE-A3, MAGE-A4, and NY-ESO-1 for mucosal melanoma patients, demonstrating significant CTL activation (38). Similarly, Lynch et al. (2021) proposed a vaccine construct targeting CEA (carcinoembryonic antigen) and Her2 peptides, a well-known CRC biomarker, showing promising immunogenic potential (39). More recently, Pant et al. (2024) developed a peptide-based vaccine targeting KRAS mutations and reported enhanced T-cell responses against pancreatic and CRC in phase 1 AMPLIFY-201 trial (40). Compared to these efforts, our study uniquely focuses on the cancer-testis antigens DKKL1, FBXO39, and OIP5, which have been largely underexplored in CRC immunotherapy despite their selective overexpression and potent immunogenic potential. By combining *in-silico* prediction, docking, molecular dynamics, and *in vitro* cytokine validation, our approach provides a comprehensive characterization of novel CTL epitopes that are conserved, non-allergenic, and capable of eliciting strong immune responses. This positions our vaccine candidates as promising additions to the current landscape of CRC immunotherapies, with the potential for high tumor specificity and minimal off-target effects.

The *in-vitro* validation demonstrated that the selected multi-epitope vaccine constructs effectively stimulated immune responses, as evidenced by significant elevations in IFN-γ, IL-2, and TNF-α levels. The dose-dependent increase in cytokine secretion indicates robust activation of CTL, reinforcing the immunogenic potential of the designed epitopes. These findings align with previous studies highlighting the role of IFN-γ and IL-2 in enhancing T-cell-mediated immunity, essential for effective vaccine responses (41). The molecular dynamics simulations were performed under standard conditions (300 K temperature and 1 atm pressure) typically used for protein-ligand interactions. However, we recognize that the tumor microenvironment, with its unique conditions (e.g., hypoxia, altered pH, and nutrient availability), could affect epitope-HLA interactions. Future simulations incorporating these factors will provide a more accurate representation of the vaccine’s behavior *in-vivo*. The low RMSD values and minimal residue fluctuations in RMSF analysis suggest strong and sustained binding affinity. The stable hydrogen bond occupancy observed throughout the simulation highlights the structural integrity of the peptide-HLA complexes, supporting their potential for efficient antigen presentation (42). Furthermore, *in-vivo* validation using mouse models of colorectal cancer will be critical to assess the tumor-targeting ability of the vaccine constructs and evaluate the induction of both humoral and cellular immune responses in a more complex biological context. These planned extensions will help to strengthen the foundation for future clinical trials, ensuring the efficacy and safety of the constructs in treating cancer. Despite the strong immunogenic potential of our vaccine, several challenges must be addressed for clinical application. HLA restriction and Inter-patient variability, These challenges highlight the need for further studies, including animal models and clinical trials, to refine the vaccine strategy.

## Conclusion

CTAs are highly promising targets for CTL-based immunotherapy in colorectal cancer due to their restricted expression in normal tissues and high immunogenicity. Their association with tumor progression and metastasis enhances their therapeutic relevance. This study leveraged computational immuno-informatics tools to identify high-affinity HLA class I-restricted epitopes from DKKL1, FBXO39, and OIP5, providing a basis for the development of a peptide-based vaccine for colorectal cancer immunotherapy. We successfully identified and validated multiple CTL-specific epitopes from DKKL1, FBXO39, and OIP5, demonstrating strong binding affinities with HLA class I alleles. The constructed multi-epitope vaccines were stable and exhibited robust immunogenic potential in-silico. Furthermore, their effective interaction with TLR-4 indicates their capability to trigger a comprehensive immune response, highlighting their potential as candidates for CTL-based immunotherapy in colorectal cancer. However, the findings are based solely on in-silico analyses, which, while valuable, may not fully replicate the complexity of the human immune system. Experimental validation, including *in-vitro* and *in-vivo* studies, is essential to confirm the immunogenicity, safety, and efficacy of the identified epitopes. Despite this limitation, the study provides a strong basis for further experimental research, paving the way for the advancement of a peptide-based vaccine for colorectal cancer.

## Data Availability

The datasets presented in this study can be found in online repositories. The names of the repository/repositories and accession number(s) can be found in the article/**Supplementary Material**.

## References

[1] Rawla P Sunkara T Barsouk A. Epidemiology of colorectal cancer: incidence, mortality, survival, and risk factors. Przegla d Gastroenterol. (2019) 14:89. doi: 10.5114/pg.2018.81072 PMC6791134 31616522

[2] Bray F Ferlay J Soerjomataram I Siegel RL Torre LA Jemal A. Global cancer statistics 2018: GLOBOCAN estimates of incidence and mortality worldwide for 36 cancers in 185 countries. CA Cancer J Clin. (2018) 68:394–424. doi: 10.3322/caac.21492 30207593

[3] Kumar A Gautam V Sandhu A Rawat K Sharma A Saha L. Current and emerging therapeutic approaches for colorectal cancer: A comprehensive review. World J Gastrointest Surg. (2023) 15:495. doi: 10.4240/wjgs.v15.i4.495 37206081 PMC10190721

[4] Simpson AJG Caballero OL Jungbluth A Chen Y-T Old LJ. Cancer/testis antigens, gametogenesis and cancer. Nat Rev Cancer. (2005) 5:615–25. doi: 10.1038/nrc1669 16034368

[5] Suri A Jagadish N Saini S Gupta N. Targeting cancer testis antigens for biomarkers and immunotherapy in colorectal cancer: Current status and challenges. World J Gastrointest Oncol. (2015) 7:492–502. doi: 10.4251/wjgo.v7.i12.492 26691579 PMC4678396

[6] Xie N Shen G Gao W Huang Z Huang C Fu L. Neoantigens: promising targets for cancer therapy. Sig Transduct Target Ther. (2023) 8:1–38. doi: 10.1038/s41392-022-01270-x PMC9816309 36604431

[7] Fan C Qu H Wang X Sobhani N Wang L Liu S. Cancer/testis antigens: from serology to mRNA cancer vaccine. Semin Cancer Biol. (2021) 76:218–31. doi: 10.1016/j.semcancer.2021.04.016 33910064

[8] Zhuang Z Zhuo J Yuan Y Chen Z Zhang S Zhu A. Harnessing T-cells for enhanced vaccine development against viral infections. Vaccines. (2024) 12:478. doi: 10.3390/vaccines12050478 38793729 PMC11125924

[9] Wagner S Mullins CS Linnebacher M. Colorectal cancer vaccines: Tumor-associated antigens vs neoantigens. World J Gastroenterol. (2018) 24:5418–32. doi: 10.3748/wjg.v24.i48.5418 PMC6319136 30622371

[10] Zhu G Song J Chen W Yuan D Wang W Chen X. Expression and role of dickkopf-1 (Dkk1) in tumors: from the cells to the patients. Cancer Manag Res. (2021) 13:659–75. doi: 10.2147/CMAR.S275172 PMC7847771 33536782

[11] Gong J Zhou Y Liu D Huo J. F-box proteins involved in cancer-associated drug resistance. Oncol Lett. (2018) 15:8891–900. doi: 10.3892/ol.2018.8500 PMC5958692 29805625

[12] Zhang X Gu W Lin A Duan R Lian L Huang Y. The role of OIP5 in the carcinogenesis and progression of ovarian cancer. J Ovarian Res. (2023) 16:185. doi: 10.1186/s13048-023-01265-4 37660035 PMC10474646

[13] Zheng Z Wieder T Mauerer B Schäfer L Kesselring R Braumüller H. T cells in colorectal cancer: unravelling the function of different T cell subsets in the tumor microenvironment. Int J Mol Sci. (2023) 24:11673. doi: 10.3390/ijms241411673 37511431 PMC10380781

[14] Fan T Zhang M Yang J Zhu Z Cao W Dong C. Therapeutic cancer vaccines: advancements, challenges and prospects. Sig Transduct Target Ther. (2023) 8:1–23. doi: 10.1038/s41392-023-01674-3 PMC10716479 38086815

[15] Patronov A Doytchinova I. T-cell epitope vaccine design by immunoinformatics. Open Biol. (2013) 3:120139. doi: 10.1098/rsob.120139 23303307 PMC3603454

[16] Wilkins MR Gasteiger E Bairoch A Sanchez JC Williams KL Appel RD. Protein identification and analysis tools in the ExPASy server. Methods Mol Biol. (1999) 112:531–52. doi: 10.1385/1-59259-584-7:531 10027275

[17] Gupta R Brunak S. Prediction of glycosylation across the human proteome and the correlation to protein function. Pac Symp Biocomput. (2002) 310–22. 11928486

[18] Larsen MV Lundegaard C Lamberth K Buus S Lund O Nielsen M. Large-scale validation of methods for cytotoxic T-lymphocyte epitope prediction. BMC Bioinf. (2007) 8:424. doi: 10.1186/1471-2105-8-424 PMC2194739 17973982

[19] Doytchinova IA Flower DR. VaxiJen: a server for prediction of protective antigens, tumour antigens and subunit vaccines. BMC Bioinf. (2007) 8:4. doi: 10.1186/1471-2105-8-4 PMC1780059 17207271

[20] Dimitrov I Bangov I Flower DR Doytchinova I. AllerTOP v.2–a server for in silico prediction of allergens. J Mol Model. (2014) 20:2278. doi: 10.1007/s00894-014-2278-5 24878803

[21] Steentoft C Vakhrushev SY Joshi HJ Kong Y Vester-Christensen MB Schjoldager KTBG. Precision mapping of the human O-GalNAc glycoproteome through SimpleCell technology. EMBO J. (2013) 32:1478–88. doi: 10.1038/emboj.2013.79 PMC3655468 23584533

[22] Kozakov D Hall DR Xia B Porter KA Padhorny D Yueh C. The ClusPro web server for protein-protein docking. Nat Protoc. (2017) 12:255–78. doi: 10.1038/nprot.2016.169 PMC5540229 28079879

[23] Heo L Park H Seok C. GalaxyRefine: protein structure refinement driven by side-chain repacking. Nucleic Acids Res. (2013) 41(Web Server issue):W384-8. doi: 10.1093/nar/gkt458 PMC3692086 23737448

[24] Rapin N Lund O Castiglione F. Immune system simulation online. Bioinformatics. (2011) 27:2013–4. doi: 10.1093/bioinformatics/btr335 21685045

[25] Chauhan V Singh MP. Immuno-informatics approach to design a multi-epitope vaccine to combat cytomegalovirus infection. Eur J Pharm Sci. (2020) 147:105279. doi: 10.1016/j.ejps.2020.105279 32119992

[26] Nezafat N Ghasemi Y Javadi G Khoshnoud MJ Omidinia E. A novel multi-epitope peptide vaccine against cancer: an in silico approach. J Theor Biol. (2014) 349:121–34. doi: 10.1016/j.jtbi.2014.01.018 24512916

[27] Fan A Wang B Wang X Nie Y Fan D Zhao X. Immunotherapy in colorectal cancer: current achievements and future perspective. Int J Biol Sci. (2021) 17:3837–49. doi: 10.7150/ijbs.64077 PMC8495390 34671202

[28] Niu J Li X-M Wang X Liang C Zhang Y-D Li H-Y. DKK1 inhibits breast cancer cell migration and invasion through suppression of β575 catenin/MMP7 signaling pathway. Cancer Cell Int. (2019) 19:168. doi: 10.1186/s12935-019-0883-1 31285694 PMC6591985

[29] González-Sancho JM Aguilera O García JM Pendás-Franco N Peña C Cal S. The Wnt antagonist DICKKOPF-1 gene is a downstream target of beta-catenin/TCF and is downregulated in human colon cancer. Oncogene. (2005) 24:1098–103. doi: 10.1038/sj.onc.1208303 15592505

[30] Wang Z Liu P Inuzuka H Wei W. Roles of F-box proteins in cancer. Nat Rev Cancer. (2014) 14:233–47. doi: 10.1038/nrc3700 PMC4306233 24658274

[31] Di Gregorio J Di Giuseppe L Terreri S Rossi M Battafarano G Pagliarosi O. Protein stability regulation in osteosarcoma: the ubiquitin-like modifications and glycosylation as mediators of tumor growth and as targets for therapy. Cells. (2024) 13:537. doi: 10.3390/cells13060537 38534381 PMC10969184

[32] Chun H-K Chung K-S Kim HC Kang J-E Kang MA Kim J-T. OIP5 is a highly expressed potential therapeutic target for colorectal and gastric cancers. BMB Rep. (2010) 43:349–54. doi: 10.5483/bmbrep.2010.43.5.349 20510019

[33] Zhu M Takano A Tsevegjav B Yoshitake Y Shinohara M Daigo Y. Characterization of Opa interacting protein 5 as a new biomarker and therapeutic target for oral cancer. Int J Oncol. (2022) 60:27. doi: 10.3892/ijo.2022.5317 35103287

[34] Nicolás-Morales ML Luisa-Sanjuan A Gutiérrez-Torres M Vences-Velázquez A Ortuño-Pineda C Espinoza-Rojo M. Peptide-based vaccines in clinical phases and new potential therapeutic targets as a new approach for breast cancer: A review. Vaccines (Basel). (2022) 10:1249. doi: 10.3390/vaccines10081249 36016136 PMC9416350

[35] Bhattacharya P Budnick I Singh M Thiruppathi M Alharshawi K Elshabrawy H. Dual role of GM-CSF as a pro-inflammatory and a regulatory cytokine: implications for immune therapy. J Interferon Cytokine Res. (2015) 35:585–99. doi: 10.1089/jir.2014.0149 PMC4529096 25803788

[36] Lotfi N Thome R Rezaei N Zhang G-X Rezaei A Rostami A. Roles of GM604 CSF in the pathogenesis of autoimmune diseases: an update. Front Immunol. (2019) 10:1265. doi: 10.3389/fimmu.2019.01265 31275302 PMC6593264

[37] Cui L Wang X Zhang D. TLRs as a promise target along with immune checkpoint against gastric cancer. Front Cell Dev Biol. (2020) 8:611444. doi: 10.3389/fcell.2020.611444 33469538 PMC7813757

[38] Freiberger SN Holzmann D Morand GB Hüllner M Levesque MP Dummer R. Combinational expression of tumor testis antigens NY-ESO-1, MAGE-A3, and MAGE-A4 predicts response to immunotherapy in mucosal melanoma patients. J Cancer Res Clin Oncol. (2023) 149:5645–53. doi: 10.1007/s00432-022-04514-z PMC10356647 36527482

[39] Lynch KT Squeo GC Kane WJ Meneveau MO Petroni G Olson WC. A pilot trial of vaccination with Carcinoembryonic antigen and Her2/neu peptides in advanced colorectal cancer. Int J Cancer. (2022) 150:164–73. doi: 10.1002/ijc.33793 34480368

[40] Pant S Wainberg ZA Weekes CD Furqan M Kasi PM Devoe CE. Lymph-node-targeted, mKRAS-specific amphiphile vaccine in pancreatic and colorectal cancer: the phase 1 AMPLIFY-201 trial. Nat Med. (2024) 30:531–42. doi: 10.1038/s41591-023-02760-3 PMC10878978 38195752

[41] Arya A Arora SK. A T-cell epitope-based multi-epitope vaccine designed using human HLA specific T cell epitopes induces a near-sterile immunity against experimental visceral leishmaniasis in hamsters. Vaccines (Basel). (2021) 9:1058. doi: 10.3390/vaccines9101058 34696166 PMC8537199

[42] Singh W Karabencheva-Christova TG Black GW Ainsley J Dover L Christov CZ. Conformational dynamics, ligand binding and effects of mutations in nirE an S627 adenosyl-L-methionine dependent methyltransferase. Sci Rep. (2016) 6:20107. doi: 10.1038/srep20107 26822701 PMC4731766

