# Supplementary material for: Design and immunogenic evaluation of multi-epitope vaccines for colorectal cancer: insights from molecular dynamics and In-Vitro studies
Source: Front Oncol. 2025 Jun 17;15:1592072. doi: 10.3389/fonc.2025.1592072 (PMC12209213; doi:10.3389/fonc.2025.1592072)
Supplement: Supplementary file 1 [file DataSheet1.docx]

**Supplementary Figure 1:** Multiple sequence alignment of target proteins. DKKL1 (A), FBXO39 (B) and OIP5 (C).

**
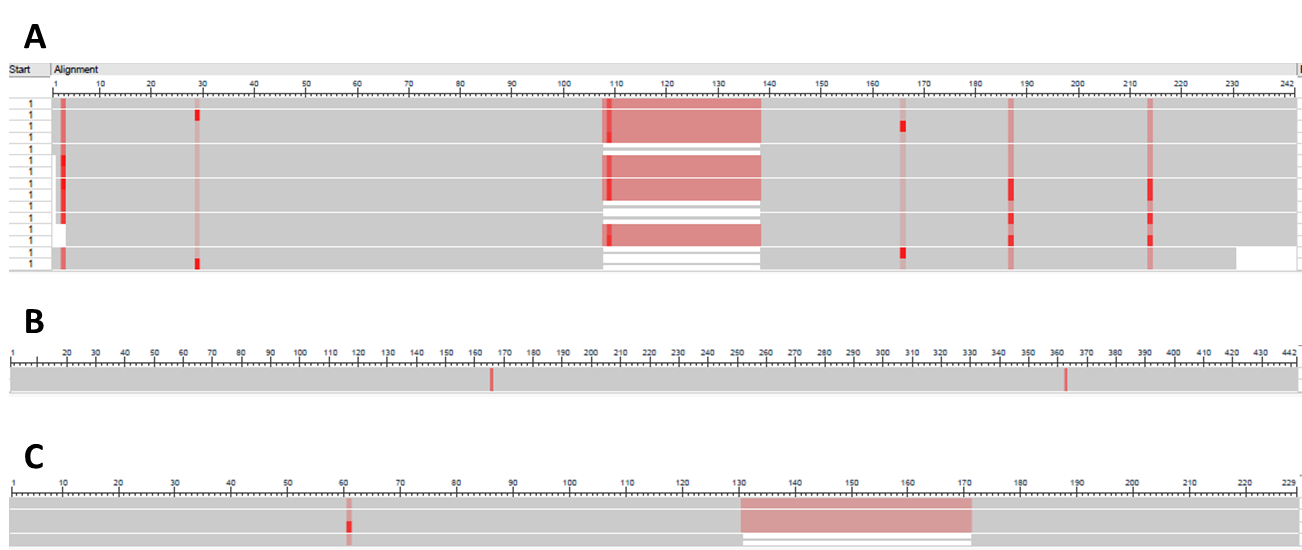
**

**Supplementary Table 1:** Physicochemical properties of the target proteins: DKKL1, FBXO39 and OIP5.

| **Physicochemical properties** | **DKKL1** | **FBXO39** | **OIP5** |
| --- | --- | --- | --- |
| Mol wt | 27006.79 | 52645.99 | 24691.45 |
| Theoretical pI | 8.10 | 9.10 | 7.02 |
| Extinction coefficients | 13980 | 85675 | 21470 |
| Estimated half-life (Escherichia coli, in vivo) | >10 hours | >10 hours | >10 hours |
| Instability index | 62.34 | 41.85 | 40.18 |
| Aliphatic index | 94.75 | 92.17 | 89.48 |
| Grand average of hydropathicity (GRAVY) | -0.45 | -0.278 | 0.010 |

**Supplementary Figure 2: Glycosylation sites in target proteins:** **DKKL1 (A), FBXO39 (B) and OIP5 (C).**

**A**


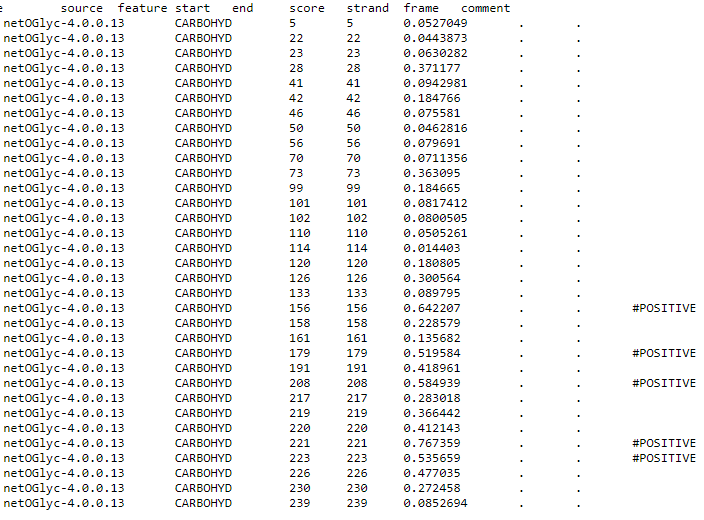


**B**


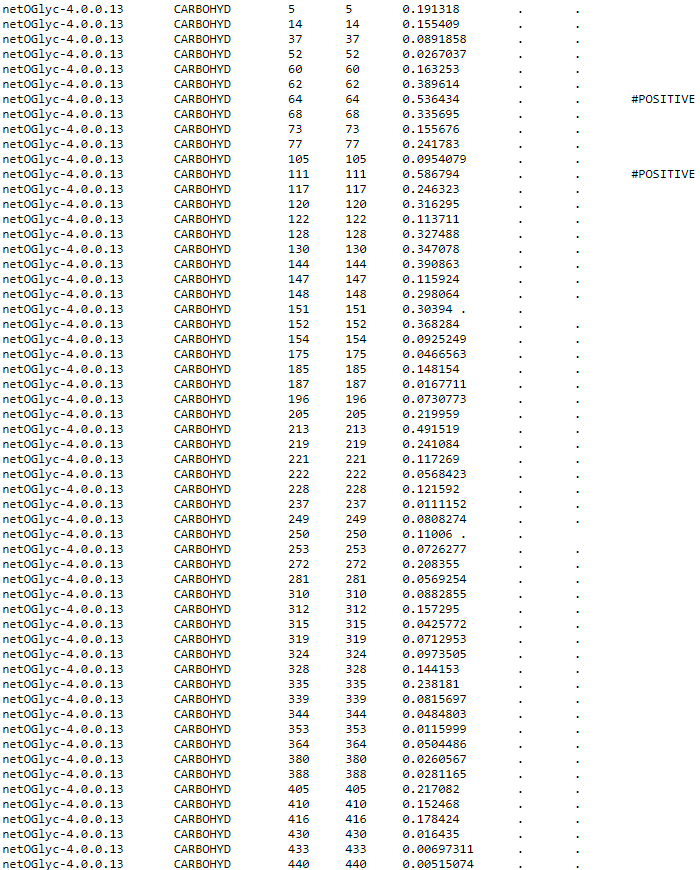


C
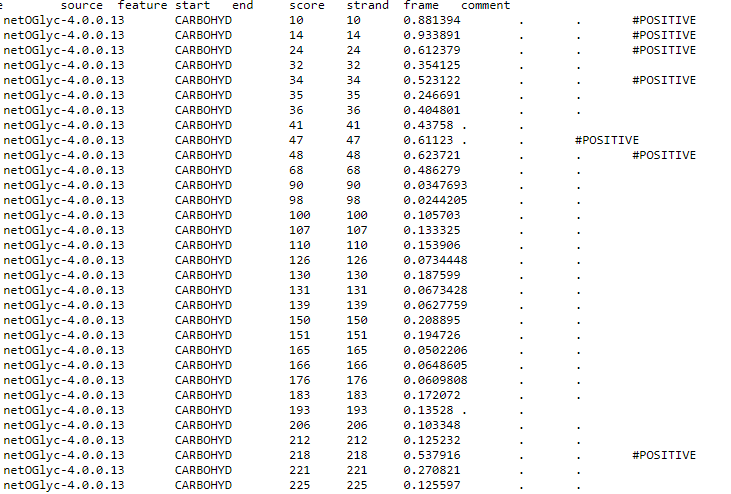


| **Suppmetery Table -2** **Cytokine Release and CD8+ T Cell Activation Summary** | | | |
| --- | --- | --- | --- |
| **Vaccine Construct** | **Cytokine Release (IFN-γ, IL-2)** | **% CD8+ T Cells Activated (CD69/CD25)** | **Proliferation Index (CD8+ Cells)** |
| Vaccine Construct 1 | IFN-γ: 120 pg/mL, IL-2: 85 pg/mL | 40% | 50% |
| Vaccine Construct 2 | IFN-γ: 130 pg/mL, IL-2: 95 pg/mL | 45% | 60% |
| Vaccine Construct 3 | IFN-γ: 110 pg/mL, IL-2: 75 pg/mL | 35% | 45% |
| Untreated Control | IFN-γ: 25 pg/mL, IL-2: 20 pg/mL | 10% | 5% |
